# No Trade-Off between Learning Speed and Associative Flexibility in Bumblebees: A Reversal Learning Test with Multiple Colonies

**DOI:** 10.1371/journal.pone.0045096

**Published:** 2012-09-20

**Authors:** Nigel E. Raine, Lars Chittka

**Affiliations:** Biological and Experimental Psychology Group, School of Biological and Chemical Sciences, Queen Mary, University of London, London, United Kingdom; University of Arizona, United States of America

## Abstract

Potential trade-offs between learning speed and memory-related performance could be important factors in the evolution of learning. Here, we test whether rapid learning interferes with the acquisition of new information using a reversal learning paradigm. Bumblebees (*Bombus terrestris*) were trained to associate yellow with a floral reward. Subsequently the association between colour and reward was reversed, meaning bees then had to learn to visit blue flowers. We demonstrate that individuals that were fast to learn yellow as a predictor of reward were also quick to reverse this association. Furthermore, overnight memory retention tests suggest that faster learning individuals are also better at retaining previously learned information. There is also an effect of relatedness: colonies whose workers were fast to learn the association between yellow and reward also reversed this association rapidly. These results are inconsistent with a trade-off between learning speed and the reversal of a previously made association. On the contrary, they suggest that differences in learning performance and cognitive (behavioural) flexibility could reflect more general differences in colony learning ability. Hence, this study provides additional evidence to support the idea that rapid learning and behavioural flexibility have adaptive value.

## Introduction

Learning gives animals the opportunity to modify their behaviour in response to changes in the environment. Results emerging in recent years support the idea that variation in learning performance appears to be linked to differences in fitness. In the laboratory, insects able to form associations between cues and predictable rewards perform better than animals prevented from learning [1], [2]. Selection experiments indicate that enhanced learning [3], [4] or long term memory performance [5] are associated with potential fitness costs in *Drosophila*. Furthermore, fast learning appears to confer a selective advantage for bumblebees colonies foraging under natural conditions [6]. All this evidence lends support to the hypothesis that animal learning and memory performance is likely to be under selection. However, if faster learning confers fitness benefits, why don't all individuals in a population display high-speed acquisition? One possibility is that there is a trade-off between rapid learning, and other memory-related performance [7], [8]. Might very rapid acquisition result in tightening of associations too quickly, at the expense of future flexibility to deal with environmental change? In an extreme form, this is illustrated in the phenomenon of imprinting, where one-trial learning can essentially result in a fixed and life-long behaviour pattern [9]. But the same question is of course equally relevant in other forms of learning [10], [11]. Reversal learning [12] is a standard experimental paradigm used to examine such cognitive/behavioural flexibility [13]–[17] because it involves either suppressing or undoing the initial association, and/or overwriting it with new (potentially conflicting) information [18], [19]. Reversal learning relies on different molecular/neural mechanisms to initial associative learning, and, at least in mammals, involves different brain regions [15], [16], [18], [20]–[23]. Here, we investigate the potential trade-off between acquisition and reversal learning using bumblebee (*Bombus terrestris*) colonies faced with an ecologically relevant associative reversal learning paradigm.

In nature, bees forage in a dynamic floral market, typically containing dozens of flowers species, which not only differ in their nectar and pollen rewards, but also their appearance, handling costs, and spatial distribution. Depending on patterns of reward production and the activities of other flower visitors, the average rewards in a flower species may change rapidly during the course of a day [24]–[26]. Thus, learning to associate which flower species are the most rewarding, and when, could have a significant impact on foraging success. Previously, we have demonstrated that variation in learning speed among bumblebee colonies is directly correlated with foraging performance, a robust fitness measure, under natural conditions [6], [27]. The slowest learning colonies collected around 40% less nectar than the fastest learning colonies, suggesting strong selection for higher learning speed. This raises the question of what maintains this appreciable intercolony variation in learning speed.

The apparent fitness costs of enhanced cognitive performance in insects [3]–[5] could create an investment trade-off between learning/memory and other essential functions (e.g. immunity). While mounting an immune response to fight an infection reduces the ability of individual bees to both form and recall a learnt association [28], [29], there is no evidence for an investment trade-off between learning and immune function at the colony level [30]. An alternative hypothesis is a potential trade-off between learning speed and memory-related performance such that rapid learning (and memory consolidation) might interfere with the acquisition of new (potentially conflicting) information [18], [31], [32]. For example, bees can learn the necessary motor skills to effectively extract rewards from multiple flower species, but task efficiency suffers if bees juggle multiple memories in a short time period [33], [34]. During acquisition, a learnt association becomes consolidated (stabilised) as a memory trace over time. Memory consolidation may occur during the initial acquisition of the association or may happen multiple times (reconsolidation) after the memory is retrieved [31], [35]. Results from honeybees (*Apis mellifera*) suggest there are differences in the cellular mechanisms of memory consolidation following initial and reversal learning [23], [36], underlining the differences in these two learning processes. A simple way to test whether rapid initial learning interferes with acquiring new (and potentially conflicting) information is a reversal learning paradigm, which involves suppressing an earlier (learned) association while a new association is formed [14], [19], [37], [38]. Here we compare variation in learning performance amongst individual workers within the same colony and among colonies. Whilst learning occurs at the individual level, bumblebee reproduction is restricted to a subset of individuals within each colony. Hence heritable intercolony (rather than inter-individual) variation in performance forms the raw material upon which any selection for learning ability could act [39]–[41]. If a trade-off exists between rapid learning and other memory-related performance, we expect faster learning colonies in the initial phase to learn more slowly than other colonies in the reversal foraging scenario.

## Materials and Methods

We obtained bumblebee (*Bombus terrestris dalmatinus*) colonies from Koppert Biological Systems (Berkel en Rodenrijs, Netherlands). Prior to experiments, bees were fed pollen and artificial nectar *ad libitum* without exposure to coloured stimuli associated with food. All workers were uniquely marked on the thorax with numbered, coloured tags (Opalith tags, Christian Graze KG, Germany). This allowed individuals to be accurately identified in laboratory learning experiments.

Controlled illumination for laboratory experiments was provided by high frequency fluorescent lighting (TMS 24F lamps with 4.3 kHz ballasts, Philips, Netherlands fitted with Activa daylight tubes, Osram, Germany) to simulate natural daylight above the bee flicker fusion frequency.

### Learning performance

#### Pre-training

Bees were pre-trained to forage from 20 bicoloured, blue and yellow, artificial flowers in a laboratory flight arena. The square, bicoloured flowers were constructed from two halves (each 12×24 mm): one yellow (Perspex® Yellow 260) the other blue (Perspex® Blue 727). During pre-training all bicoloured flowers were rewarded with 50% (w/w) sucrose solution providing previously colour-naïve bees with an equal chance to associate both colours with reward [6], [27]. Bees completing at least 5 consecutive foraging bouts on bicoloured flowers were selected for training.

Results from a pilot study indicate that variation in the number of pre-training bouts, beyond this threshold of 5 consecutive foraging bouts, does not significantly affect the speed with which bees subsequently learn to associate yellow as a predictor of reward. The learning performance of 20 bees (from a single colony) was assessed using the same paradigm as the initial training phase in experiment 2 (see below). Individual bees varied in the number of pre-training bouts they performed (range = 5–24 bouts) prior to training. The number of pre-training bouts performed by a bee was not significantly correlated with subsequent learning speed (*t* value) during training (when yellow flowers were rewarding and blue flowers were empty: Spearman's rank correlation coefficient (r_s_) = −0.270, n = 20, p = 0.249).

#### Experiment 1: Inter-individual variation in learning performance

Foragers were trained individually in a flight arena containing 15 blue (Perspex® Blue 727) and 15 yellow (Perspex® Yellow 260) artificial flowers (each 24×24 mm). During the first phase of training (initial learning), yellow flowers were most rewarding (each contained 10 µl of 50% (w/w) sucrose solution), whilst blue flowers contained lower concentration rewards (10 µl of 25% (w/w) sucrose solution). We recorded the choice sequence made by each bee from the time it first entered the flight arena, until it made at least 100 flower choices (over at least two consecutive foraging bouts), including the first time it probed a more rewarding (yellow) flower, plus any choices made before this first probing event. In all cases this resulted in the bee reaching saturation performance on the initial learning task.

The following morning we tested overnight memory retention of the initial phase of the learning task with an unrewarded choice test. Each test bee was observed during a single foraging bout in the flight arena containing 15 blue and 15 yellow unrewarded artificial flowers. During this bout we recorded the number of times the test bee chose each flower colour from which we could calculate its learned colour preference for yellow.

Following the overnight memory retention test, we reversed the association between flower colour and reward (reversal learning): therefore, in this second training phase, blue flowers were most rewarding (each contained 10 µl of 50% (w/w) sucrose solution), whilst yellow flowers contained lower concentration rewards (10 µl of 25% (w/w) sucrose solution). We recorded all flower choices made by each bee (following the reversal of rewarding flower colour) until it made at least 100 flower choices including the first time it probed a blue (more rewarding) flower in the second training phase (plus any choices made before this first probing event). Hence, each bee made at least 200 flower choices in total, i.e. at least 100 choices in each of the two, initial (day 1) and reversal (day 2), training phases. In total we tested 18 bees from a single colony in this experiment.

#### Experiment 2: Intercolony variation in learning performance

The general training procedure for this experiment was similar to that described for experiment 1. Foragers were trained individually, in a flight arena containing 10 blue and 10 yellow artificial flowers. During the first phase of training (initial learning), yellow flowers were rewarding (each contained 15 µl of 50% (w/w) sucrose solution), whilst blue flowers were empty (completely unrewarding). Each bee was observed until it made at least 100 flower choices, including the first time it probed a rewarding (yellow) flower. Upon completion of the initial learning phase of training, we immediately reversed the association between flower colour and reward (reversal learning): therefore, in this second training phase, blue flowers were rewarding (each contained 15 µl of 50% (w/w) sucrose solution), and yellow flowers were now unrewarding (empty). Hence initial and reversal phases of the learning task were conducted on the same day (meaning that overnight memory retention of the association of yellow as a predictor of reward learned during the initial training phase could not be assessed). We recorded all flower choices made by each bee (following the reversal of rewarding flower colour) until it made at least 100 flower choices, including the first time it probed a rewarding (blue) flower (plus any choices made before this first probing event). Hence, each bee made at least 200 flower choices in total, i.e. at least 100 choices in each of the initial and reversal training phases.

Fifteen bees were trained from each of six colonies (i.e. 90 bees in total) of which 80 completed both training phases (of the 10 bees that failed to complete reversal training 6 failed to probe a blue (rewarding) flower and 4 ceased foraging before completing a sufficient number of flower choices). In both experiments flowers were changed and their positions re-randomized between foraging bouts to prevent bees using scent marks or previous flower positions as predictors of reward. Flower colours were selected so that bees had to overcome their innate preference for blue [42], [43], before associating yellow (one of their innately least favourite colours) with reward during the initial training phase. Bees were then challenged to reverse this association in the reversal training phase. Some earlier studies suggest a correlation between bumblebee worker body size and learning and memory performance [44], [45], although we have not found such a correlation in our work [27]. Nonetheless, because body size is correlated with sensory performance in some tasks [46], [47], thorax width measurements were taken for each test bee as a measure of body size.

Learning data were collected simultaneously from multiple colonies, with observers moving haphazardly between colonies when foragers were ready for training (i.e. when bees choose to participate in the paradigm). Hence, while there will always be some minor variation in conditions (e.g. time of day) when each bee was tested our approach should not have introduced any systematic (consistent) differences among colonies in variables (at least partially) outside experimenter control. This view is supported as we see no significant difference among colonies in the average time of day when training started (Table 1a). All colonies began this experiment at a similar age/developmental stage and we ensured they all had equal access to food throughout the experimental period. We found no significant variation among colonies in the average number or duration of bouts performed in either the initial or reversal training phases (Table 1b–e). While minor variation in ‘uncontrolled parameters’ is inevitable, even under laboratory conditions, this actually enhances the ecological relevance of our results since when foraging in the field bees are learning in the face of significantly greater variation in environmental conditions.

**Table 1:** Intercolony variation in seven training parameters for experiment 2.

Colony	D3	D4	D6	D8	D9	D10	Kruskal-Wallis (*X* ^2^) p-value
	mean ± S.E. (median)						
a) Start time of training	11:57±00:25 (11:40)	12:52±00:35 (12:29)	14:07±00:07 (14:12)	13:46±00:39 (13:52)	13:01±00:34 (13:11)	12:44±00:28 (13:11)	(10.00) 0.075
**Initial Learning**							
b) Number of bouts	4.13±0.35 (4)	4.87±0.39 (5)	3.8±0.09 (4)	3.73±0.42 (3)	3.67±0.23 (4)	3.73±0.53 (3)	(7.52) 0.185
c) bout duration/secs	246.7±17.5 (260)	267.8±26.5 (235)	287.9±9.8 (260)	354.7±61.3 (234)	237.6±17.6 (222)	271.1±34.7 (253)	(2.53) 0.771
**Reversal Learning**							
d) Number of bouts	4.27±0.49 (4)	5.73±0.57 (6)	4.93±0.51 (5)	5.14±0.40 (5)	4.47±0.36 (4)	4.67±0.65 (5)	(5.52) 0.356
e) bout duration/secs	251.3±26.9 (246)	260.0±26.6 (241)	212.0±20.3 (190.5)	244.5±26.7 (207.5)	227.1±14.6 (195)	224.7±24.1 (199)	(4.90) 0.428
**Number of Landings**							
f) yellow lands (initial)	29.9±2.0 (28)	37.4±2.8 (33)	25.3±2.2 (27)	31.4±2.2 (30)	36.1±2.5 (36)	30.9±2.7 (29)	(12.41) **0.029**
g) blue lands (reversal)	32.1±3.1 (35)	45.7±4.0 (43)	38.0±3.4 (40.5)	42.1±2.7 (43.5)	44.0±3.2 (42)	38.7±4.0 (38)	(7.72) 0.173

(a) The average time when training started for bees in each of the six colonies. The average number (b) and duration (c) of bouts performed by bees in the initial training phase. The average number (d) and duration (e) of bouts performed by bees in the reversal training phase. The average number of landings made by a bee on rewarding flowers, yellow landings during initial training (f) and blue landings in reversal training (g), during the 100 flower choices (including the first time it probed a rewarding flower for the first time). In all cases the mean (± S.E.) and median values are given for each colony. The results from Kruskal-Wallis tests indicate whether variation among colonies is significant (statistically significant p-values are shown in bold).

### Fitting learning curves

**Figure 1:**
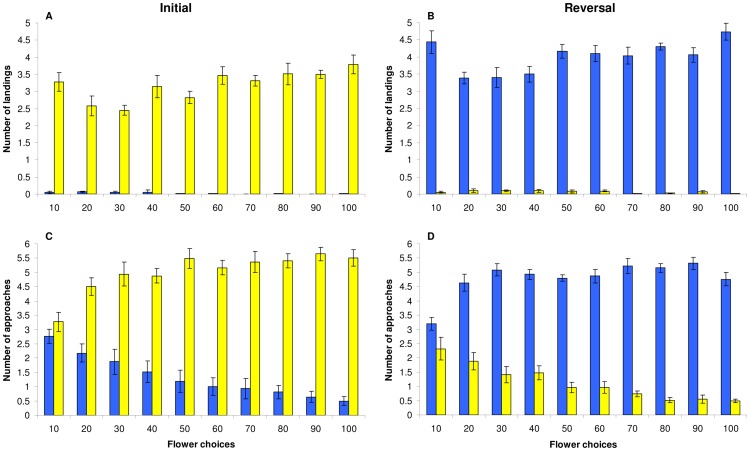
Summary of all flower choices made by foragers in the initial and reversal phase of experiment 2. Choices are broken down into the colony mean (±1 S.E.) numbers of blue and yellow landings (panels A and B) and approaches (panels B and D) made during consecutive bins of 10 flower choices (n = 6 colonies). The flower choices begin with the first time the bee fed from a rewarding flower (yellow in the initial and blue in reversal phase).

In both experiments, bees were regarded as choosing a flower when they either approached (inspected), or landed on it (although landing on a flower did not necessarily result in a feeding (probing) event). Approach (inspection) flights have been found to be informative as indicators of floral choice in our paradigm, since we found that bumblebees increased the frequency of both approach flights and landing events to the (more) rewarding flower colour with increasing individual experience (see Figure 1, [48]).

Bees are highly sensitive to the sugar concentration of nectar and will choose more concentrated nectar when it is available [37], [49], [50]. Hence, choosing the most rewarding (experiment 1)/sole rewarding (experiment 2) flower colour was regarded as ‘correct’, whilst choosing a less rewarding (experiment 1)/totally unrewarding (experiment 2) flower colour was deemed to be an ‘error’ (the colour of correct choice changed between the initial and reversal learning phases of training).

**Figure 2:**
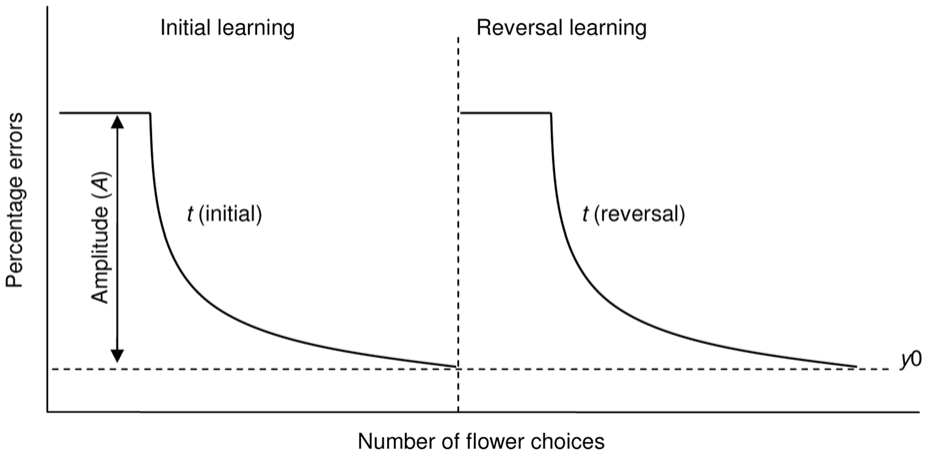
Schematic diagram illustrating how bee performance changes during the initial and reversal phases of the learning task. Here, the percentage of errors (less rewarding (experiment 1) or unrewarding (experiment 2) flowers chosen) is plotted against number of flower choices made by a hypothetical bee. The initial learning phase (during which yellow flowers are (more) rewarding) is shown in the left hand panel, whilst the reversal learning phase (during which blue flowers are now (more) rewarding) is shown on the right hand side. The dashed vertical line indicates the point at which the association between floral colour and rewards are reversed. The bee starts the initial learning phase with an innate preference for blue (over yellow), hence initially chooses a high percentage of blue (less rewarding or unrewarding) flowers. Once the bee probes a (more) rewarding, yellow flower the percentage of blue flowers chosen begins to drop as it learns to associate yellow as a predictor of floral rewards. The rate of performance improvement is initially fast, before gradually levelling off to the final task performance plateau (*y_0_*). Bees return to making a high percentage of errors when the association between flower colour and reward are reversed. The yellow flowers they learned to visit in the initial learning phase are now less rewarding/totally unrewarding. As soon as bees probe a blue flower, which now contains (more) rewards, they receive positive reinforcement that this colour is now (more) rewarding.

Two learning curves were fitted to the flower choice data for each individual bee to capture the dynamic nature of the associative learning process in both the initial and reversal phases of training. In each case the starting point for each learning curve was the percentage of errors made (less rewarding or unrewarding flowers chosen) before the bee first probed a (more) rewarding flower for the first time (Figure 2). For bees making fewer than 5 flower choices (either by approaching or landing on them) before probing a rewarding flower (n = 0 of 18 bees experiment 1; n = 17 of 90 (19%) initial phase and 8 of 80 (10%) reversal phase respectively experiment 2), we used the colony mean percentage of errors (calculated from bees making at least 5 such choices). Flower choices made by each bee after (and including) the first time it probed a (more) rewarding flower were evaluated as the number of errors (less rewarding or unrewarding flowers chosen) in each group of 10 choices. Learning curves (first order exponential decay functions: *y = y_0_+Ae^−x/t^*) were fitted to these eleven data points (i.e. the starting point and subsequent 10 groups of ten flower choices) for each individual bee, using Microcal Origin® [6]. This was repeated twice for each bee, once for the initial phase in which yellow flowers were (more) rewarding, and again for the reversal phase in which blue flowers were (more) rewarding. In both cases, *x* is the number of flower choices made by a bee, starting with the first time it probed a (more) rewarding flower, and *y* is the number of errors (i.e. number of less rewarding or unrewarding flowers chosen). The saturation performance level (*y_0_*) is the number of errors made by a bee after finishing the learning process, i.e. when reaching a performance plateau. The decay constant (*t*) is a measure of learning speed: high values of *t* correspond to slow learning, whereas lower *t* values indicate faster learners. *A* is the curve amplitude: the maximum displacement (height) of the curve above *y_0_* (Figure 2). Both amplitude (*A*) and saturation performance (*y_0_*) were constrained between 0–10 for curve fitting.

## Results

### Experiment 1: Inter-individual variation in learning performance

**Figure 3:**
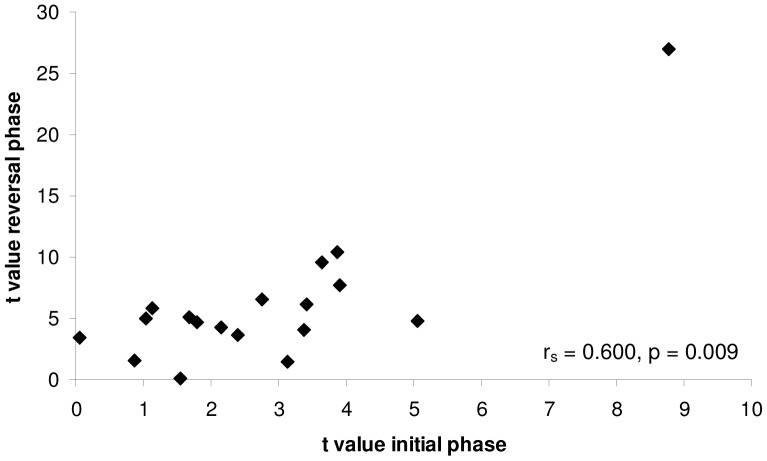
Correlation between initial and reversal learning speed for eighteen bumble-bee workers from a single colony. High *t* values correspond to slow learning, while low values are generated by fast learners. Each data point corresponds to the learning speed (*t* value) for an individual bee. On average, workers which learnt faster (had lower *t* values) in the initial learning task were also faster at learning to reverse this colour association (r_s_ = 0.600, n = 18, p = 0.009). This correlation remains significant even if the outlying data point on the right hand side of the figure is excluded (r_s_ = 0.525, n = 17, p = 0.031).

Individual bees from the same colony showed appreciable and predictable variation in learning performance during both phases of this experiment. We found a significant positive correlation between the speed with which an individual learnt to associate yellow as the most rewarding colour in the initial phase and the speed with which they learned to associate blue as a predictor of higher rewards in the reversal phase (r_s_ = 0.600, n = 18, p = 0.009: Figure 3). On average, bees which were quick when learning to associate yellow as a predictor of higher reward in the initial phase, were also fast at learning that blue was a good predictor of higher rewards in the reversal phase (low *t* values for both phases of the experiment).

**Figure 4:**
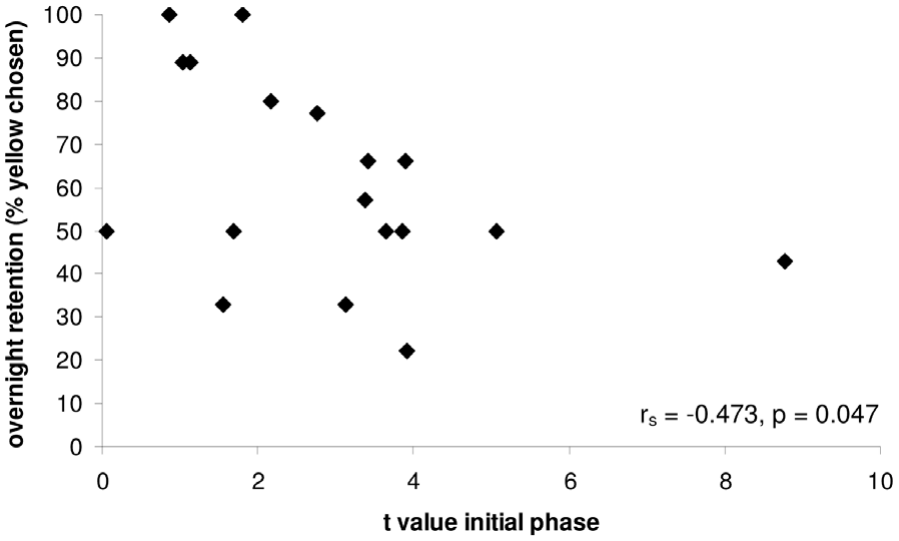
Correlation between initial learning speed and overnight retention of learned association for eighteen bumble-bee workers from a single colony. Bees which were quick to learn to associate yellow as a predictor of high levels of floral reward have low *t* values. Overnight retention of this learned association was assessed by recording the percentage of yellow flowers chosen in an unrewarded choice test with both blue and yellow flowers (see Methods for details). On average, workers which learnt more quickly that yellow was a predictor of higher concentration sucrose solution rewards (had lower *t* values) in the initial learning phase were also likely to show a stronger learned preference for yellow in the overnight retention test (r_s_ = −0.473, n = 18, p = 0.047).

Faster learning individuals in the initial phase also retain the learnt association in memory better than slower learners. Workers that were quicker to learn to associate yellow as a predictor of higher rewards in the initial training phase (i.e. those with low *t* values) also showed stronger overnight retention of this learned colour association (r_s_ = −0.473, n = 18, p = 0.047: Figure 4). However, the performance of bees in the unrewarded overnight memory retention test was a very poor predictor of their learning speed in the reversal training phase (r_s_ = 0.009, n = 18, p = 0.974). This shows that a) ‘forgetting’ the initially learnt association overnight was not a prerequisite for faster reversal learning; and b) visiting more flowers of the previously rewarded colour during the unrewarded retention test did not predispose bees to reverse learn faster.

When comparing the performance of individual bees within the same colony we see no evidence of a trade-off between the speed of initial learning and either the subsequent ability to acquire new information or the reliability of memory retrieval (rather both these factors are positively correlated with initial learning speed). In addition, these data indicate that choices for the less rewarding flower colour are indeed ‘errors’, rather than the bee exploring alternatives to gather information: if this was not the case we would expect that bees making more errors in the initial phase should learn faster in the reversal phase, which is the opposite of what was observed.

### Experiment 2: Intercolony variation in learning performance

**Figure 5:**
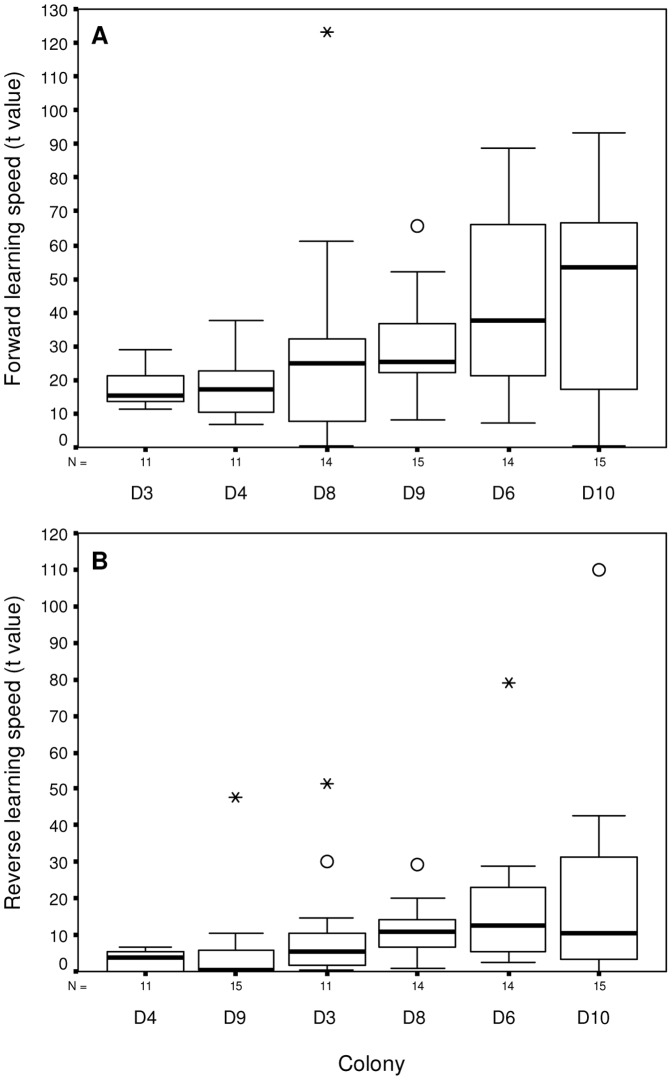
Variation in learning speed (*t* values) of bumblebees from the six colonies in the initial and reversal learning phase of experiment 2. High values of *t* correspond to slow learning bees, whereas lower *t* values indicate faster learners. In each box the thick horizontal bar is the colony median, whilst the lower and upper edges represent the 25% and 75% quartiles respectively. Whiskers indicate the maximum and minimum values that are not outliers. Outliers are represented by open circles, extreme values by asterisks. The number of bees tested in each colony (N) is displayed along the x-axis, and colonies are ranked by increasing 75% quartile values from left to right. Variation in learning speed for the initial phase is shown in panel A, and for the reversal phase in panel B.

There was significant variation in colony learning speed in both the initial and reversal phases of the learning task (*t* value: Kruskal-Wallis: *X* ^2^ = 14.283, p = 0.014 (initial) and *X* ^2^ = 21.67, p = 0.001 (reversal); Figure 5). The differences in learning speed between bees in these colonies were highlighted when we compared the number of flower choices taken to reduce the number of errors made by 80% from starting performance towards their saturation level (*y_0_*, i.e. move 80% of the way from the top to the bottom of their learning curve). In the initial phase bees from the fastest learning colony (D3) took on average only 29 flower visits to achieve an 80% improvement in task performance (from starting error levels), while bees from the slowest learning colony (D10) took 105 visits to reach the same performance level (therefore, these two colonies differed in learning speed by a factor of 3.6). In the reversal phase, bees from the fastest learning colony (D4) took on average only 5 flower visits to achieve an 80% improvement in task performance (from starting error levels), while bees from the slowest learning colony (D10) took 33 visits to reach the same level of performance (therefore, these two colonies differed in learning speed by a factor of 7.2).

Although there was also significant variation among colonies in the number of rewarding (yellow) flowers bees landed on during the initial training phase (Kruskal-Wallis: *X* ^2^ = 12.417, p = 0.029: Table 1f), this was not significantly correlated with (*t* value) learning speed (r_s_ = −0.257, n = 6, p = 0.623) or other measures of learning performance. There was no significant intercolony variation in the average number of rewarding (blue) flowers bees landed on during the reversal training phase (Kruskal-Wallis: *X* ^2^ = 7.715, p = 0.173: Table 1g).

**Figure 6:**
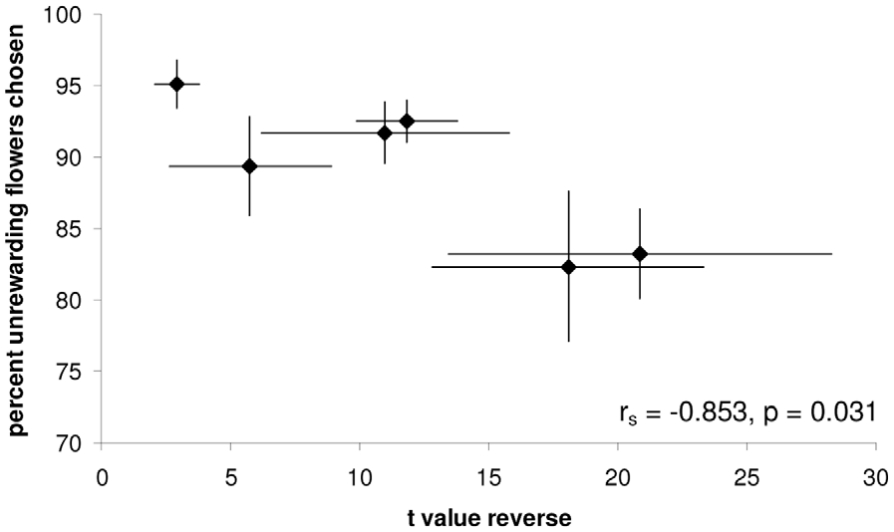
Correlation between percentage errors before probing first rewarding (blue) flower and learning speed for six colonies in reversal phase. High *t* values correspond to slow learning, while low values are generated by fast learners. Data presented are colony mean (±1 S.E.) *t* values on the x-axis, and the mean (±1 S.E.) percentage of unrewarding, yellow flowers chosen by each colony on the y-axis. On average, colonies which made more errors before probing a rewarding, blue, flower for the first time also had higher learning speed in the reversal phase of this learning task (r_s_ = −0.853, n = 6, p = 0.031).

We found a significant negative correlation between colony *t* value and percentage of unrewarding (yellow) flowers chosen before probing a rewarding flower in the reversal phase (r_s_ = −0.853, n = 6, p = 0.031: Figure 6). This suggests that colonies which choose yellow more frequently (before probing blue) in the reversal task also have higher learning speed (lower *t* values). This correlation remained significant when controlling for significant intercolony variation in average forager size (thorax width: Kruskal-Wallis: *X* ^2^ = 20.464, p = 0.001) with partial correlation (partial correlation coefficient = −0.8894, p = 0.043).

**Figure 7:**
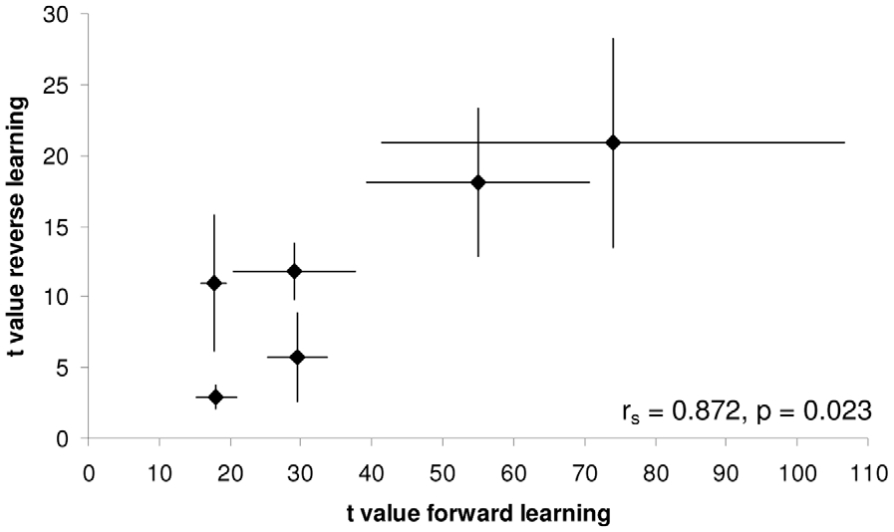
Correlation between initial and reversal learning speed for six bumble-bee colonies. High *t* values correspond to slow learning, while low values are generated by fast learners. Data presented are colony mean *t* values (±1 S.E.). On average, colonies with higher learning speeds (lower *t* values) in the initial learning task were also faster at learning to reverse this colour association (r_s_ = 0.872, n = 6, p = 0.023).

Comparing the average colony performance we found a significant positive correlation between colony learning speed (*t* value) in the initial and reversal learning phase (r_s_ = 0.872, n = 6, p = 0.023; Figure 7). Controlling for significant intercolony variation in average forager size (thorax width), this correlation between initial and reversal learning speed was still upheld (partial correlation coefficient = 0.8941, p = 0.041). Thus colonies which were fast at learning to associate yellow as a predictor of reward in the initial phase were also quick to learn in the reversal situation.

## Discussion

Our study relates to a fundamental question in the evolutionary biology of learning – why is learning gradual rather than instantaneous [10], [11]? We examine the potential trade-offs between rapid learning and other memory-related performance using an ecologically relevant associative learning paradigm. If variation in the speed with which an association is learned has significant repercussions for subsequent behavioural flexibility, we would expect the learning performance of initially rapid learners to be subsequently impaired when associations (such as those between floral colour and reward) are reversed. Here, we find a positive correlation between the learning speed of both individuals within a single colony (experiment 1), and also among colonies (experiment 2), in their performance in the initial and reversal phases of a colour learning task. This suggests that both at the individual and colony level fast initial learning does not appear to constrain subsequent cognitive flexibility. Overall, our results provide no evidence of a trade-off between learning speed and memory performance in bumblebees in this visual associative learning task, but indeed the opposite.

Whilst the process of learning happens within the brain of an individual bee, reproduction in social insects is restricted to a subset of individuals within each colony. Hence heritable intercolony, rather than inter-individual, variation in cognitive performance forms the raw material upon which any selection for learning ability might act. However, before we consider the potential adaptive consequences of variation in cognitive flexibility at the colony level, we must first consider the evidence for trade-offs in individual workers. Comparing the performance of individual bees (experiment 1), our results support the idea that workers which were fast learners in the initial phase were also quicker to reverse this association; this contrasts with the hypothesis of a trade-off between initial learning speed and subsequent cognitive flexibility (at least within the same sensory modality). Increasing interest in the behavioural syndrome perspective [51]–[53], suggests this angle deserves further direct investigation. Indeed, more consistent relative performance of individuals might be observed across contexts if learning ability was assessed across different sensory modalities rather than two visual tasks as used in our experiments. In the field of human research this interest in consistency of ‘intelligence’ across tasks dates back well over 100 years, and is the very philosophy underpinning IQ tests [54]–[56].

Decision accuracy is dependent on the information available to the animal making the choice. Gathering additional information, or improving the quality of the information already available, typically improves decision accuracy. However it usually incurs a cost in terms of the time invested to obtain it [57]. If information, such as which flower species currently contains the most rewards, can quickly become inaccurate due to changes in the environment animals may adopt behavioural strategies to update the information they have. One possible strategy for foraging bees could be to make periodic exploratory visits to each different flower species to check what rewards they contain [24], [58], [59]. Hence, it is possible that bees in our experiments may have chosen flowers containing lower quality rewards to evaluate whether the information about the relative rewards of both flower colours they had learnt was still correct. In such a scenario these choices for the less rewarding flower colour would not be an error in their associative learning, but potentially an adaptive choice. If bees choosing the less rewarding colour were indeed gathering information we would expect bees making more such choices to perform better in the reversal learning phase. However, our results provide no support for this idea suggesting that choices for the less rewarding colour are indeed decision errors.

We also observed that faster learning individuals in the initial phase were better at retaining this learnt association in memory overnight than slower learners. Interestingly, this finding contrasts with work on *Drosophila* larvae indicating that the rover genotype is quicker at learning to avoid a conditioned odour than the sitter genotype, but that rovers were poorer than sitters at retaining this learned association [7], [8], although the *Drosophila* research compared the performance of two distinct genotypes with a single gene polymorphism, and our study documented variation among bumblebee workers within a colony.

Although lasting only a single foraging bout, the overnight memory retention test (in which both flower colours were unrewarding) could have lead to partial extinction of the initial learned association (in experiment 1). However, even in the absence of such an unrewarded overnight retention test, the overall effect (a positive correlation between the initial and the reversal learning phase) was the same in experiment 2. It is also important to keep in mind that extinction (on a per trial basis) is a much slower process than acquisition (i.e. many more extinction trials are needed to achieve the same change in behavioural response as in rewarded trials [60], [61]) - in other words a brief unrewarded phase is unlikely to have a profound effect on subsequent reversal learning, especially since it was experienced by all individuals equally. Comparable reversal learning protocols to experiment 1 have been used in other studies (e.g. [62]), although because they trained bees in groups using proboscis extension reflex (PER) conditioning it is not possible to elucidate any differential effects of extinction trials (between initial and reversal learning phases) on individual bees.

The overnight memory retention test might have differential effects on bees depending on their performance in the initial training task. Bees with better overnight memory could visit yellow more frequently during the retention test, allowing them more opportunity to extinguish the initial association, potentially making them better prepared to undertake reversal training. If this hypothesis is correct we would expect that overnight retention performance should predict reversal learning speed. However this is not the case - the performance of bees in the unrewarded overnight memory retention test was very poorly correlated with their learning speed in reversal training. So while the initial learning speed of individual bees predicts both their overnight retention performance and reversal learning speed, individual overnight retention performance does not predict reversal learning speed.

**Figure 8:**
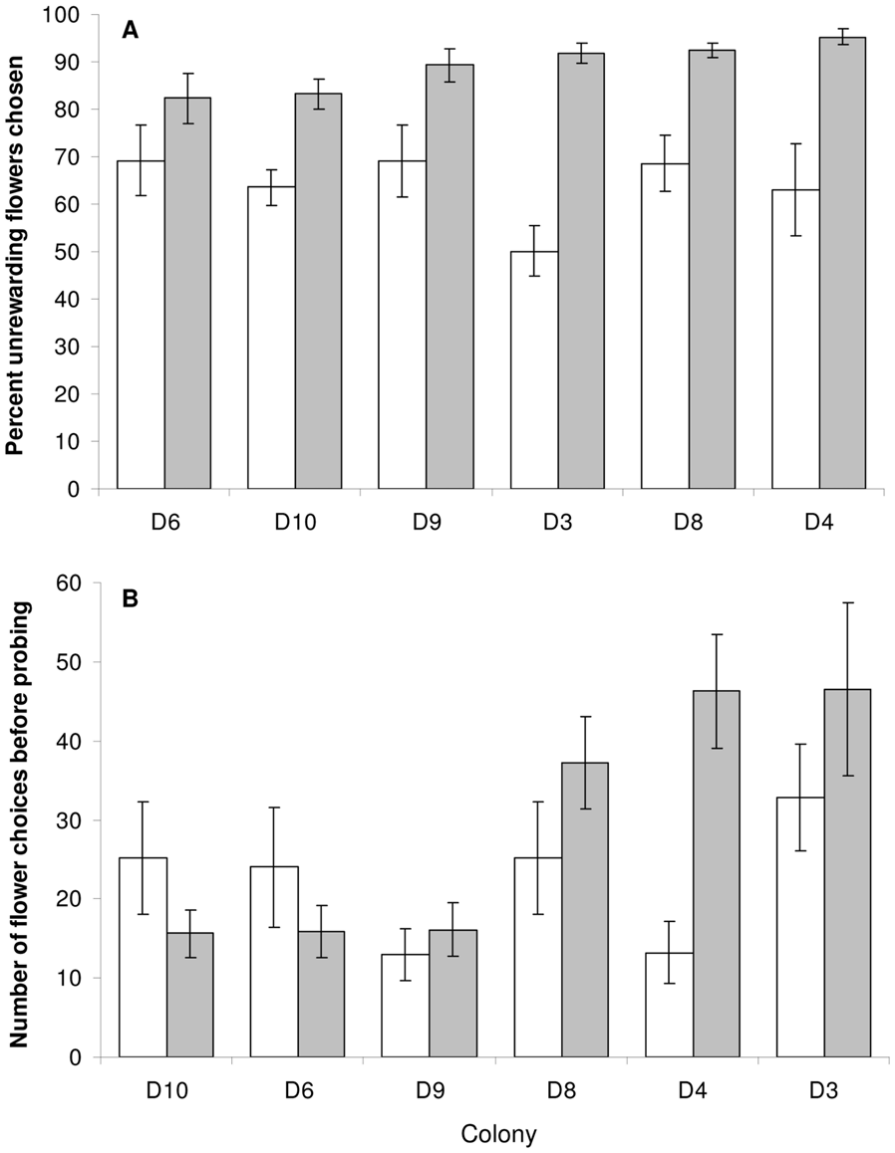
Flower choices made before probing a rewarding flower for the first time in both the initial and reversal phase of experiment 2. In the initial learning phase, there were no significant intercolony differences in either the percentage of unrewarding (blue) flowers chosen, effectively the strength of preference for blue over yellow (Kruskal-Wallis: *X* ^2^ = 6.965, p = 0.222: white columns – panel A) or the number of flower choices made before probing a rewarding (yellow) flower for the first time (Kruskal-Wallis: *X* ^2^ = 7.735, p = 0.171: white columns – panel B). Hence, on average all bees chose blue flowers 64.3% (±2.7: mean ±1 S.E.) of the time, and made 22.1 (±2.6: mean ±1 S.E.) flower choices before they probed a rewarding (yellow) flower for the first time. When the association between colour and reward was reversed, we observed intercolony variation in the percentage of unrewarding (yellow) flowers chosen before probing a rewarding flower (Kruskal-Wallis: *X* ^2^ = 10.341, p = 0.066), although non-significant, this variation among colonies is suggestive of a trend (colony mean range = 82.3–95.2%: grey columns – panel A). There was significant intercolony variation in the number of flower choices made before probing a rewarding (blue) flower (Kruskal-Wallis: *X* ^2^ = 27.532, p<0.0005: grey columns – panel B). Three colonies made on average only 15 or 16 choices, whilst the other three colonies made between 37 and 47. Column heights are colony mean (±1 S.E.) values. Colonies are ordered left to right by increasing percentage (panel A) or number (panel B) of unrewarding (yellow) flowers chosen in the reversal phase.

Comparing mean *t* values for the initial and reversal tasks for each colony indicates that members of all colonies learned the reverse association (between blue and reward) considerably more quickly than the initial association between yellow and reward (initial phase: Figure 7). Whilst all bees have more experience learning in this particular context (arena cues, etc.) by the time the reversal is performed, we might have expected this result because naïve *B. terrestris* workers show a strong innate bias for blue over yellow in unrewarded choice tests [42], [43]. Hence, during the initial phase bees must overcome their innate preference for blue (over yellow) and learn to associate yellow as a predictor of floral reward. In this experiment all colonies showed an initial preference for blue prior to probing a rewarding, yellow flower for the first time (overall mean across 6 colonies = 64.3%: Figure 8a). This initial blue preference was effectively modified by experience during the initial learning phase, meaning that bees began the reversal learning phase with a strong learned preference for yellow (colony mean range = 82.3–95.2%: Figure 8a). It is interesting that despite the fact that this yellow preference at the start of the reversal phase is considerably stronger than the blue preference at the onset of initial learning, the learning speed of each colony was appreciably faster in the reversal (compared to initial) phase. Also, those colonies which chose yellow more frequently, prior to probing a blue, rewarding flower for the first time, had higher average learning speed in the reversal phase. This suggests that stronger initial colour bias, whether learned or innate, promotes more rapid association of the initially non-preferred colour and reward. Another possible explanation why bees learned the reverse association more quickly than the initial association might be related to the overlearning reversal effect [63], [64]; when training continues beyond the task saturation level this ‘overtraining’ (overlearning) can lead to the animal showing a greater readiness for reversal learning [13], [65].

Evidence from honeybees suggests that their associative learning performance deteriorates significantly following serial reversal of stimulus-reward contingencies with either two colours [64] or odours [38]. This suggests that serial reversals of same pair of stimuli (whether odours or colours) cause honeybees to struggle with the discrimination task (whether free-flying [64] or harnessed [38]). Another study suggesting honeybee learning performance actually improved with exposure to serial successive reversals between odour cues and reward [66] could be explained by configural learning as the odour pairs to be discriminated in each phase of the reversal training procedure were unique (e.g. phase 1: A+ vs. B−, phase 2: B+ vs. C−, phase 3: C+ vs. D− (+ = rewarded, − = unrewarded odours) [38]). It would be of interest to examine if bumblebees respond in a similar way if trained in serial reversal experiments in the laboratory. Evidence from *Bombus impatiens* trained to turn left or right in a T-maze depending on the colour presented at the maze entrance suggests that after a period of relatively poor task performance, learning can improve after seven or more reversals [32].

It is easy to see how both fast initial learning and subsequent behavioural flexibility, by rapid reversal of learned associations, might be advantageous to a bee foraging in a complex environment in which the predictive value of floral cues changes rapidly. As bumblebee colonies in our study that learned to associate yellow with rewards rapidly were also quick to reverse this association, this suggests fast learning does not compromise subsequent flexibility (at least when considering visual learning tasks). This ability to rapidly learn to make and break associations between floral colour and reward is likely to have contributed to the higher levels of nectar foraging efficiency (a robust proxy measure of colony fitness) shown by faster learning *B. terrestris* colonies in our earlier study [6]. As all colonies experienced very similar environmental conditions (both in commercial rearing facilities and during their time in the laboratory) we infer that the variation in learning performance observed at both the individual and colony level is largely genetically determined. Due to reproductive division of labour, any selective forces on cognitive performance will act primarily on heritable variation at the colony level. However, the similar correlation between initial learning speed and subsequent behavioural flexibility both among individuals (within a colony) and also among colonies suggests that selection could also be indirectly affecting individual performance (e.g. via pleiotropic effects).

Our results indicate that some colonies are better able to learn to form and reverse associations between colour and reward. This might suggest that colony differences in learning performance and flexibility could reflect more general differences in colony cognitive ability, or ‘general intelligence’ (*g*) [54], [56]. It would be interesting to examine whether colonies which learn (and reverse) colour associations rapidly also show consistently high levels of learning performance in other visual tasks (e.g. spatial learning) or in associative tasks involving other sensory modalities (e.g. odour or tactile cue learning). Preliminary support for this view comes from honeybee learning experiments (using proboscis extension response conditioning) in which the group of individuals which were most sensitive to sucrose stimuli show improved learning in both odour and tactile conditioning [50], [67]. If future work can confirm that performance levels in an associative learning task using one modality are indeed indicative of relative performance in other modalities across individuals and colonies we would be closer to the important goal of understanding the adaptive value of variation in cognitive abilities.
